# Protective Effect of Polyphenols Extract of Adlay (*Coix lachryma-jobi* L. var. *ma-yuen Stapf*) on Hypercholesterolemia-Induced Oxidative Stress in Rats

**DOI:** 10.3390/molecules17088886

**Published:** 2012-07-26

**Authors:** Lifeng Wang, Jing Sun, Qida Yi, Xuefeng Wang, Xingrong Ju

**Affiliations:** 1 School of Food Science and Technology, Jiangnan University, Wuxi, Jiangsu 24122, China; Email: wanglifeng_8@163.com; 2 School of Food Science and Engineering, Nanjing University of Finance and Economics, Nanjing, Jiangsu 210046, China; Email: yi.qida@yahoo.com.cn (Q.Y.); wangxuefeng2870@126.com (X.W.); 3 Key Laboratory of Functional Dairy, College of Food Science and Nutritional Engineering, China Agricultural University, Beijing 100083, China; Email: sjing324@yeah.net

**Keywords:** adlay, polyphenols, hypercholesterolemia, lipid peroxidation, antioxidant enzymes

## Abstract

The present study examines the effect of polyphenols extract of adlay (*Coix lachryma-jobi* L. var. *ma-yuen Stapf*) (APE) on high cholesterol diet fed rats (HCD). APE was orally administrated by gavage at doses of 10, 40 and 200 mg total phenolics/kg body weight of rats once a day for 28 days. At the end of four weeks, serum triglyceride (TG), total cholesterol (TC), low density lipoprotein cholesterol (LDL-C) and high density lipoprotein cholesterol (HDL-C), and markers of oxidative stress *viz*., malondialdehyde (MDA), superoxide dismutase (SOD), catalase (CAT) and glutathione peroxidase (GSH-Px) in the serum and liver of HCD and normal rats were assessed and compared. The results showed that administration of APE was significantly effective in decreasing the serum levels of TC, LDL-C and MDA, increasing the serum level of HDL-C and antioxidant capacity. In addition, oral gavage of APE could also increase the antioxidant capacity, CAT and GSH-Px activities in liver. These results suggested that APE exerted a high hypocholesterolemic and antioxidant activities, which might be characterized by a protective effect on cardiovascular health *in vivo*.

## 1. Introduction

Coronary heart disease (CHD), which is closely associated with atherosclerosis, is a major cause of death in developed countries. One of the initial events in the development of atherosclerosis is the accumulation of cells contained excess lipids within the arterial wall. Hypercholesterolemia, especially elevated level of serum cholesterol and low-density lipoprotein (LDL), has been implicated in the initiation of atherosclerosis [1]. Furthermore, oxidative stress is also suggested as a mechanism underlying hypercholesterolaemia, which is an important etiologic factor in atherosclerosis [2]. According to the oxidative modification hypothesis, oxidation of LDL is crucial to the cellular uptake of LDL in the first stages of atherosclerotic plaque development [3]. Currently, lowering level of serum lipid and enhancing the antioxidant capacity can be carried out via medication. Although chemical drugs are characterized by good efficacy, they cannot meet the demands to all hyperlipidemia patients because of the potential adverse effects. Compared with medication, plant products are generally considered to be less toxic and less prone to side effect, and have been receiving more and more attention in recent years [4].

Adlay (*Coix lachryma-jobi* L. var. *ma-yuen Stapf*), also called Job’s tears or Chinese pearl barley, is an annual crop which mainly planted in India, Japan and China [5]. Adlay has been consumed in orient countries as a nutritional food, as well as a traditional Chinese medicine which is traditionally used for wart treatment, rheumatism, neuralgia, promoting digestion, diuretic and inflammatory treatment [6]. Several physiological functions of adlay and its biological active components in different part of adlay, including hull, bran, testa and endosperm, have been investigated recently. Chen *et al.* found flavonoids in adlay bran partly contribute to its anti-inflammatory effect [7]. Chung *et al*. investigated the antiulcer activity of dehulled adlay, and demonstrated that caffeic acid was one of the compounds indicative of a gastroprotective agent [8]. They also demonstrated the ethyl acetate fraction of adlay bran ethanolic extract retard carcinogenesis through an anti-inflammatory pathway, and potential active component was ferulic acid [9]. However, few studies reported the hypocholesterolemic activity of adlay polyphenols and how it regulates the antioxidant defense system *in vivo*. Therefore, our study aimed to evaluate its hypocholesterolemic and antioxidant effects on high cholesterol diet fed rats.

## 2. Results and Discussion

### 2.1. Phytochemical in Adlay Phenolic Extract (APE)

The content of total phenolics and total flavonoids of APE were determined in order to evaluate the exposures in different groups. Our data showed the total phenolic and total flavonoid contents of APE were 20 mg gallic acid equivalent (GAE)/g APE, and 12.3 mg catechin equivalent (CE)/g APE, respectively.

The total phenolic content reported in our study was higher than previously reported by Choi *et al*. [10]. The different result between two studies was mainly due to the extraction method, since the method we used could extract both free and bound phytochemicals, not just the free fractions. In addition, bound phytochemicals are resistant to stomach and small intestine digestion and may reach the colon to release phytochemicals after the fermentation by colon bacteria, which may partially be responsible to the health benefits of whole grain consumption that lowering the risk of colon cancer [11]. Therefore, using the method to extract total phenols was not only for a higher content, but also for the potential beneficial functions of the bound phytochemicals. Furthermore, our results of the total flavonoid in adlay was higher than those reported in the previous studies using the AlCl_3_ method [7], which measured only partial flavonoids. The data indicated that flavonoids was one of major phytochemicals, and might be partially responsible to the potential health-promoting effects of adlay. 

According to the American (2010) and Chinese (2007) dietary guidelines [12,13], the daily recommended intake for grain approximately range from 170 g to 400 g. If all grain we consumed was whole-grain, that means we would intake 129–304 mg total phenolics from adlay per day, since the total phenolic content in adlay was 76.04 mg GAE/100 g dry weight. Then converting it to an animal model, it was equal to 10–30 mg total phenolics/kg body weight. Thus, in the present study, we used 40 mg total phenolics/kg body weight as a middle-dose, and investigated the beneficial effects of a lower dose—10 mg, and a higher dose—200 mg total phenolics/kg body weight, and the dose-dependent manner among them.

### 2.2. Effect of APE on the Body Weight Gain, Food Intake, and Liver Weight of Rats

As shown in Table 1, throughout the four week experiment, the food intake and feed efficiency of rats in all groups had been stabilized at about 30 g/d and 16%, and no significant changes were observed. Furthermore, there were no significant differences in the body weight gain (132–142 g), liver weight (11.86–13.19 g), and liver index at the end of the experiment among five groups. The results could be supported by the previous study [14], suggesting that APE did not cause serious toxicity in rats.

**Table 1:** Body weight gain, food intake, feed efficiency, liver weight, and liver index of rats.

Group	Body weight gain (g)	Food intake (g/d)	Feed efficiency (%)	Liver weight (g)	Liver index
Control	136 ± 19	31.45 ± 1.39	16.03 ± 2.18	11.86 ± 1.22	2.82 ± 0.20
Chol	134 ± 32	31.24 ± 2.41	15.93 ± 3.77	12.48 ± 1.84	2.99 ± 0.20
Chol/LAPE	132 ± 24	30.87 ± 1.15	15.79 ± 2.93	12.33 ± 0.95	2.96 ± 0.12
Chol/MAPE	142 ± 24	31.91 ± 0.97	16.48 ± 2.80	13.19 ± 1.50	3.10 ± 0.11
Chol/HAPE	141 ± 32	29.89 ± 1.76	17.44 ± 3.91	12.32 ± 1.51	3.04 ± 0.10

Control, basal diet; Chol, high-cholesterol diet; Chol/LAPE, Chol + 10 mg total phenolics/kg body weight of rats; Chol/MAPE, Chol + 40 mg total phenolics/kg body weight of rats; Chol/HAPE, Chol + 200 mg total phenolics/kg body weight of rats. Feed efficiency = body weight gain (g/d) × food intake^−1^ (g/d)^−1^ × 100%. Liver index = liver weight (g) × body weight gain^−1^ (g)^−1^ × 100.

### 2.3. Effect of APE on Serum Lipid Profile of Rats

**Figure 1:**
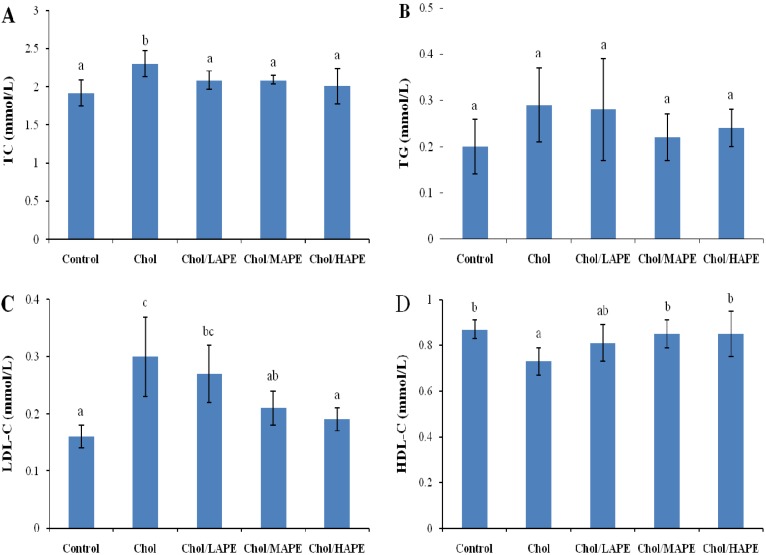
Effect of APE on serum TC (**A**), TG (**B**), LDL-C (**C**) and HDL-C (**D**) levels in rats. Values are expressed as mean ± SD. Bars not sharing common letter superscripts are significantly different (*p* < 0.05).

The *in vivo* hypocholesterolemic activity of APE was studied, and the serum lipid parameters of rats among all groups at the end of the trial were summarized in Figure 1. After four weeks of feeding, the rats in the Chol group had higher total cholesterol (TC) (2.3 ± 0.17 mmol/L *vs*. 1.92 ± 0.17 mmol/L, *p* < 0.05) and low density lipoprotein cholesterol (LDL-C) values (0.3 ± 0.07 mmol/L *vs*. 0.16 ± 0.02 mmol/L, *p* < 0.05) compared with those in the Control group. These significant increases were accompanied by lower high density lipoprotein cholesterol (HDL-C) values (0.73 ± 0.06 mmol/L *vs*. 0.87 ± 0.04 mmol/L, *p* < 0.05). However, the higher level of TC was hindered significantly (*p* < 0.05) in all APE-treated rats compared with those in the Chol group. As for LDL-C level, dose-dependent reduction were observed in the Chol/LAPE, Chol/MAPE and Chol/HAPE group (10%, 30%, and 36.67%, respectively), when compared with the Chol group. While we also noticed that the HDL-C level in the Chol/MAPE and Chol/HAPE group were significantly increased by 16.44% (*p* < 0.05), as compared to Chol group.

Many researchers have proved that the increased levels of TC and LDL-C raise the risk of developing atherosclerosis and CHD [1,15,16]. On the contrary, raised level of HDL-C was associated with reduced risk of atherosclerosis, since high density lipoprotein in serum is thought to facilitate the translocation of excess cholesterol from the peripheral tissue to liver for further catabolism [17]. In the present study, rats fed with a high-cholesterol diet for 28 days had significantly higher concentrations of serum TC and LDL-C, as well as lower concentration of HDL-C, when compared with the rats maintained on a basal diet. However, oral gavage of APE dramatically hindered the increases of serum TC and LDL-C, and improved the concentration of HDL-C with a dose-dependent manner. Especially, the reduction of TC and LDL-C were 12.61% and 36.37%, respectively, associated with a 16.44% increase of HDL-C in rats fed on a high-cholesterol diet when orally treated with 200 mg total phenolics/kg body weight of rats. According to the references studied the hypocholesterolemic effect of phenolic compounds from other sources, we suspected the reduction of TC induced by APE might due to decrease of cholesterol absorption and biosynthesis and increase of faecal bile acid and cholesterol excretion [18]. 

### 2.4. Effect of APE on Lipid Peroxidation of Hypercholesterolemic Rats

**Figure 2:**
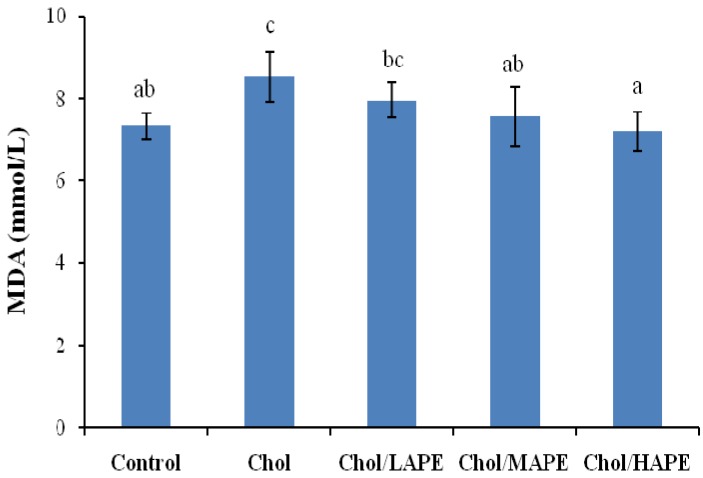
Effect of APE on serum MDA level in rats. Values are expressed as mean ± SD. Bars not sharing common letter superscripts are significantly different (*p* < 0.05).

As shown in Figure 2, the serum malondialdehyde (MDA) level of the rats in the Chol group showed marked increase (8.52 ± 0.61 mmol/L *vs*. 7.33 ± 0.31 mmol/L, *p* < 0.05) compared with rats in the Control group. However, rats in Chol/MAPE and Chol/HAPE group experienced significantly (*p* < 0.05) less of rise in the serum MDA level as compared to Chol group. MDA is a main product of lipid peroxidation, as a biomarker of oxygen free radicals, it has the potential not only to evaluate the extent of oxidative injury, but also to predict the potential efficiency of therapeutic strategies aimed at restricting the oxidative stress [19]. Previous study demonstrated that a decrease in lipid peroxidation lead to the reduction of atherosclerosis caused by hypercholesterolaemia [20]. In present study, the content of serum MDA in rat fed with high-cholesterol diet was elevated significantly compared with the rats fed with basal diet, suggesting that hypercholesterolemia might enhance the process of lipid peroxidation. It could be explained by the finding that excess cholesterol in platelets, polymorphonuclar cells, leukocytes and endothelial cells could lead to the generation of reactive oxygen species (ROS) and speeding up the course of lipid peroxidation [21]. Our data showed that administration of APE significantly decreased the content of serum MDA and suppressed the lipid peroxidation. 

### 2.5. Effect of APE on the Antioxidant Status of Hypercholesterolemic Rats

**Figure 3:**
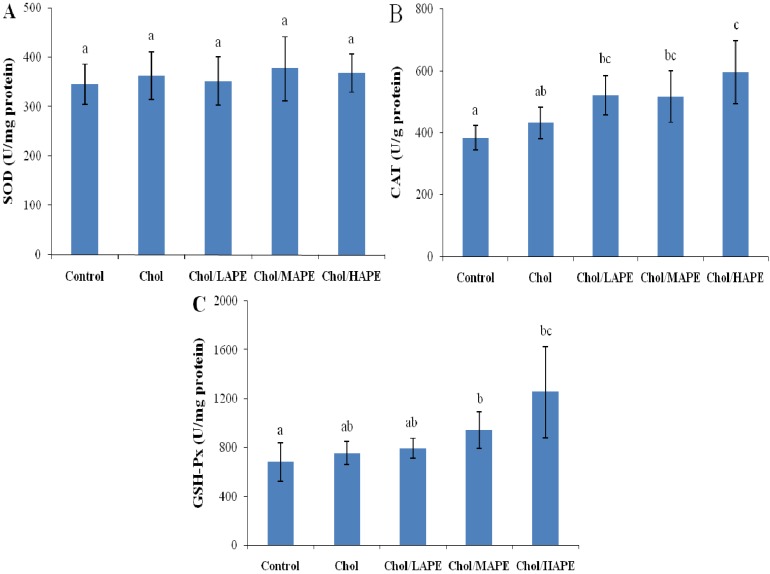
Effect of APE on hepatic SOD, CAT, and GSH-Px activities in rats. Values are expressed as mean ± SD. Bars not sharing common letter superscripts are significantly different (*p* < 0.05).

The superoxide dismutase (SOD), catalase (CAT) and glutathione peroxidase (GSH-Px) activities of liver were monitored, and the results are shown in Figure 3. There were no significant changes (*p* > 0.05) in the SOD, CAT and GSH-Px activities of liver between the Control group and Chol group. However, rats orally treated with APE at doses of 10, 40 and 200 mg total phenolics/kg body weight resulted in a dose-dependent significant rises (*p* < 0.05) in CAT (20.52%, 19.63% and 37.90%, respectively) and GSH-Px activities of liver (4.8%, 24.59% and 66.41%, respectively), as compared to the Chol group. 

**Figure 4:**
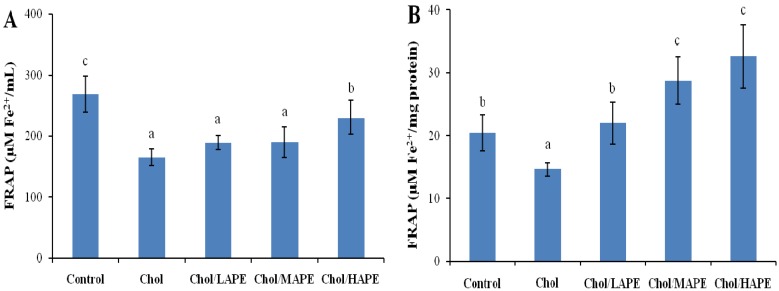
Effect of APE on antioxidant capacities of serum (**A**) and liver (**B**) in rats. Values are expressed as mean ± SD. Bars not sharing common letter superscripts are significantly different (*p* < 0.05).

The antioxidant capacities in serum and liver decreased significantly (164.97 ± 13.29 μM Fe^2+^/mL *vs*. 268.48 ± 29.25 μM Fe^2+^/mL; 14.66 ± 1.02 μM Fe^2+^/mg protein *vs*. 20.47 ± 2.86 μM Fe^2+^/mg protein, *p* < 0.05) in the Chol group compared to those in Control group (Figure 4). 

In contrast, oral gavage of APE significantly enhanced the antioxidant capacity of serum by 1.40-fold (*p* < 0.05) in the Chol/HAPE group compared with the Chol group. Nevertheless, there were no significant changes in the Chol/LAPE and Chol/MAPE compared with the Chol although the FRAP value tended to increase. Similarly, antioxidant capacity of liver in the Chol/LAPE, Chol/MAPE and Chol/HAPE group produced 1.50-fold, 1.96-fold and 2.22-fold increase (*p* < 0.05) when compared to the Chol group.

It had been mentioned that high-cholesterol diet might cause the generation of ROS, and the biological effects of ROS were controlled *in vivo* by enzymatic defense mechanisms. As an index for the redox status after four weeks with different treatments, the antioxidant capacities in serum and liver homogenates were determined. Our results showed that high-cholesterol diet might lead to significantly reduction in antioxidant capacities, whereas orally treated with APE could significantly increase the serum and hepatic antioxidant capacities in rats. In the enzymatic defense mechanism, SOD, CAT and GSH-Px are regarded as three primary antioxidant enzymes since they play important role in scavenging toxic intermediates of incomplete oxidation *in vivo* [22]. SOD catalyzes dismutation of superoxide anions into hydrogen peroxide, which was converted to water by both CAT and GSH-Px. Nutrient antioxidants, included in the dietary antioxidants, are chain breaking antioxidants, which work with enzyme antioxidants, to regular the ROS within physiological limits [23]. Our data demonstrated that treated with APE remarkable increased CAT and GSH-Px activities in rats fed with high-cholesterol diet. Phytochemicals, especially the phenolic compounds in fruits and vegetables, have been proposed as the major bioactive compounds increasing antioxidant potential *in vivo*, but data in the literature showed contradictory results on the effect of dietary phenolics on the activity of antioxidant enzymes in experimental animals or human subjects. On one hand, in parallel with our results, Yazdanparast *et al*. found that treatment of hypercholesterolaemic rats with *N. officinale* extract significantly enhanced CAT and SOD activities in liver, which were accompanied with a significant decrease in hepatic MDA [24]. Zou *et al*. reported that a flavonoid-rich extract of *Hypericum perforatum* L. increased the activity of SOD in liver and serum, as well as the activity of CAT in liver [25]. On the other hand, Han *et al*. found that hepatic GSH-Px and CAT activities were decreased by orally treated with anthocyanin-rich potato flakes in hypercholesterolaemic rats, while no significantly change was observed in hepatic SOD activity [26]. Our results might be explained by the finding that an induction of antioxidant enzymes could reflect an improvement in cellular protection, making sure that excess oxidants could be rapidly metabolized and eliminated. 

## 3. Experimental

### 3.1. Chemicals

Sodium borohydride, chloranil, catechin hydrate, Folin-Ciocalteu reagent, gallic acid and 2,4,6-tripyridyl-*S*-triazine (TPTZ) were purchased from Sigma-Aldrich, Inc. (St. Louis, MO, USA). Tetrahydrofuran and aluminum chloride were purchased from Fisher Scientific (Fair Lawn, NJ, USA). Other chemicals were purchased from Mallinckrodt Chemicals (Phillipsburg, NJ, USA). Kits for triglyceride (TG), TC, LDL-C and HDL-C were purchased from BioSino Biotechnology and Science Co., Ltd. (Beijing, China). SOD kit, CAT kit, GSH-Px kit and MDA kit were purchased from the Nanjing Jiancheng Bioengineering Institute (Nanjing, China). BCA protein assay kit was purchased from Pierce Biotechnology Inc. (Rockford, IL, USA). 

### 3.2. Preparation of APE

Adlay used in this study were purchased from local farmers who planted Longyi No.1 of *Coix lachryma-jobi* L. var. *ma-yuen Stapf* in Longyan City, Fujian Province. All adlay samples were dehusked on a Satake Rice Milling Machine (Satake Co., Hiroshima, Japan) and milled into flour by passing through a 60-mesh sieve on a Cyclone Sample Mill (UDY Corp., Fort Collins, CO, USA), then stored at −20 °C until analysis. Total phenolic compounds including free and bound phenols of adlay were extracted by the modified method reported previously [27]. In Brief, adlay flour was first digested with of 2 M sodium hydroxide (1:5, w/v) at room temperature for 1 h with shaking under nitrogen. Then, the mixture was adjusted to pH 2 with an appropriated amount of concentrated hydrochloric acid and extracted with hexane to remove lipids. Next, the final solution was extracted five times with ethyl acetate, and the ethyl acetate fractions were pooled and concentrated under reduced pressure by a rotary vacuum evaporator at 45 °C. Finally, the APE was stored at −20 °C for further analysis. 

### 3.3. Determination of the Total Phenolic and Total Flavonoid Content

The total phenolic content of APE was determined using the Folin-Ciocalteu colorimetric method described by Singleton *et al*. [28]. Gallic acid was used as the standard, and total phenolic content was expressed as mg GAE/g APE. The total flavonoid content of APE was determined using the sodium borohydride/chloranil-based assay described in previous study [29], and results were expressed as mg CE/g APE. 

### 3.4. Animals and *in Vivo* Study Design

Forty male Wistar rats weighing 200 ± 10 g (Vital River Laboratories Co., Ltd., Beijing, China) were used for the present investigation. Animals were maintained under standard conditions (23 ± 2 °C, relative humidity 55 ± 5%, 12 h light-dark cycle), and had *ad libitum* access to the diets and distilled water throughout the study. Animal maintenance and experimental procedures were approved by the Animal Ethics Committee of Nanjing University of Finance and Economics.

Prior to experimental study, animals were fed basal diet for one week for adaptation. The composition of basal diet was showed in Table 2, and it was regarded as normal diet, while the high-cholesterol diet was formulated as 99% (w/w) basal diet supplemented with 1% cholesterol (w/w). The cholesterol batches were mixed carefully with the basal diets just before the diets were offered to the rats [30]. Afterwards, animals were randomly divided into five groups with eight animals each and received the following treatments: basal diet (Control group); high-cholesterol diet (Chol group); high-cholesterol diet + 10 mg total phenolics/kg body weight of rats (Chol/LAPE group); high-cholesterol diet + 40 mg total phenolics/kg body weight of rats (Chol/MAPE group) and high-cholesterol diet + 200 mg total phenolics/kg body weight of rats (Chol/HAPE group). Dietary intake was measured daily and body weight was recorded every six days. Aqueous suspensions of APE were treated with sonication and vigorous vortex prior to administration. All the suspensions (2 mL) were administered by gavage once a day. For keeping the bioactivities of APE, the suspensions must be prepared daily.

**Table 2:** Composition of animal diets.

Ingredients	Basal diet (g/kg)	High-cholesterol diet (g/kg)
Casein	182	180
Soybean oil	61	60
Wheat starch	687	680
Cholesterol	0	10
Vitamin mixture	10	10
Mineral mixture	60	60

Vitamins (per kg of diet): thiamin, 20 mg; riboflavin, 15 mg; pyridoxin, 10 mg; nicotinamide, 100 mg; calcium panthotenate, 70 mg; folic acid, 5 mg; biotin, 0.3 mg; cyanocobalamin, 0.05 mg; retinyl palmitate, 1.5 mg; dl-a-tocopheryl acetate, 125 mg; cholecalciferol, 0.15 mg; menadione, 1.5 mg; ascorbic acid, 50 mg; myo-inositol, 100 mg; carrier wheat starch, 1.36 g. Minerals (per kg of diet): CaHPO_4_, 15 g; K_2_HPO_4_, 2.5 g; KCl, 5 g; NaCl, 5 g; MgCl_2_, 2.5 g; Fe_2_O_3_, 2.5 mg; Mn_2_SO_4_, 125 mg; CuSO_4_·7H_2_O, 0.2 mg; ZnSO_4_·7H_2_O, 100 mg; KIO_3_, 0.4 mg.

After four weeks on the experimental diets, the animals were fasted for 16 h. The next day, after recording the body weight, blood samples were collected by cardiac puncture under diethyl ether anesthesia. Serum were obtained by centrifugation 3,000 g for 15 min at 4 °C, and stored immediately at −80 °C until analysis. Then the animals were sacrificed and the livers were removed, weighed and stored immediately at −80 °C for further analysis.

### 3.5. Serum Lipids Assay

The levels of serum TC, TG, LDL-C and HDL-C were evaluated using commercially available kits according to the instructions of the manufacturer with 7020 Clinical Analyzer (Hitachi, Tokyo, Japan).

### 3.6. Lipid Peroxidation and Antioxidant Profiles Analysis

The serum MDA concentration was determined by kit using thiobarbituric acid reactive substance assay, which was based on the reaction of MDA with thiobarbituric acid to generate a colored product which can be measured at 532 nm with UV-2102 spectrophotometer (Unico Instruments Co., Ltd., Shanghai, China). 

For determination of liver antioxidant enzyme activities, liver homogenates were prepared using cold saline (0.9% NaCl) and tissue homogenizers in an ice bath, and then centrifuged at 3,000 g for 15 min, finally the supernatants were obtained for biochemical analysis. The enzyme activities, including T-SOD, GSH-Px and CAT were estimated by the test kits according to the manufacturers’ instructions. T-SOD activity was measured through an adaptation of the method of McCord and Fridovich [31]. The xanthine/xanthine oxidase system was used to generate the superoxide anion. This anion produced the reduction of cytochrome c, which was monitored at 550 nm. The SOD activity in the sample removed the superoxide anion and produced an inhibition of the cytochrome c reduction. The determination of GSH-Px activity is based on the oxidation of reduced glutathione by GSH-Px. CAT activity was determined by following the decomposition of H_2_O_2_ measured as a decrease in absorbance at 240 nm. As the optical density values of the liver samples were obtained, activities of T-SOD, GSH-Px and CAT were calculated by the respective formulas and methods provided by the kits. The amount of total protein in the supernatant of liver homogenate was determined using BCA protein assay kit, T-SOD and GSH-Px activities were expressed as active unit per mg of protein, while CAT activity was expressed as active unit per g of protein.

The antioxidant capacity of serum and liver homogenates were analyzed by the ferric reducing/antioxidant power (FRAP) assay [32]. The antioxidant activity was evaluated by measuring the samples’ ability to reduce ferric tripyridyltriazine (Fe^3+^-TPTZ) to ferrous tripyridyltriazine (Fe^2+^-TPTZ) complex, which has an intense blue color monitored at 593 nm. Briefly, 20 μL of serum samples or liver homogenates were mixed on a 96-well plate with 200 μL of FRAP reagent. Samples were incubated for 10 min at 37 °C and then absorbance at 593 nm was recorded on a microplate reader (Bio-Rad, Hercules, CA, USA). FRAP values of derived from triplicate analyses were expressed as micromole ferrous iron per mL for antioxidant capacity of serum, and micromole ferrous iron per mg of protein for antioxidant capacity of liver homogenates. 

### 3.7. Statistical Analysis

Results are expressed as means ± standard deviations (SD). Significant differences among the groups were determined by one-way ANOVA with Duncan’s multiple range tests. Differences were considered significant at *p* < 0.05. All statistical analyses of data were performed using SPSS 17.0 (SPSS, Inc., Chicago, IL, USA).

## 4. Conclusions

The present study clearly suggested that orally administration of adlay polyphenols extract exhibited a hypocholesterolemic action, inhibited formation of lipid peroxidation products, and enhanced the activities of antioxidant enzymes and antioxidant capacities of serum and liver in hypercholesterolemic rats. The results indicated that dietary supplementation with adlay is likely to reduce the risk of coronary heart disease related to hypercholesterolemia and oxidative stress.
